# Local cefuroxime tissue concentrations in the hand after single and repeated administration to 16 patients undergoing trapeziectomy: a randomized controlled trial

**DOI:** 10.2340/17453674.2024.41343

**Published:** 2024-09-06

**Authors:** Andrea René JØRGENSEN, Pelle HANBERG, Mats BUE, Charlotte HARTIG-ANDREASEN, Nis Pedersen JØRGENSEN, Maiken STILLING

**Affiliations:** 1 Aarhus Denmark Microdialysis Research Group (ADMIRE), Orthopaedic Research Unit, Aarhus University Hospital, Aarhus; 2 Department of Clinical Medicine, Aarhus University, Aarhus; 3 Department of Orthopaedic Surgery, Aarhus University Hospital, Aarhus; 4 Department of Infectious Diseases, Aarhus University Hospital, Aarhus, Denmark

## Abstract

**Background and purpose:**

The duration of antibiotic coverage in hand tissues during surgery is unknown. We investigated the time the free concentration of cefuroxime was above the minimal inhibitory concentration (*f*T>MIC) of 4 μg/mL in hand tissues after single and repeated administration.

**Methods:**

In a prospective, unblinded randomized study 16 patients (13 female, age range 51–80 years) underwent trapeziectomy. Microdialysis catheters were placed in the metacarpal bone (primary effect parameter), synovial sheath, and subcutaneous tissue. Patients were randomized to postoperative administration of either intravenous single administration of cefuroxime (1,500 mg) (Group 1, n = 8) or repeated dosing (2 x 1,500 mg) with a 4 h interval (Group 2, n = 8). Samples were taken over 8 h.

**Results:**

The *f*T>MIC of 4 μg/mL was found to be significantly longer in the metacarpal bone in Group 2 compared with Group 1 with a mean difference of 199 min (95% confidence interval 158–239). The same trend was evident in the remaining compartments. A concentration of 4 μg/mL was reached in all compartments in both groups within a mean time of 6 min (range 0–27 min). In Group 1, the mean concentrations decreased below 4 μg/mL between 3 h 59 min and 5 h 38 min.

**Conclusion:**

The *f*T>MIC was longer after repeated administration compared with single administration in all compartments. A single administration of cefuroxime 1,500 mg provided antimicrobial hand tissue coverage for a minimum of 3 h 59 min. Cefuroxime administration in hand surgeries should be done minimum 27 min prior to incision to achieve sufficient coverage in all individuals. Cefuroxime readministration should be considered in hand surgeries lasting longer than 4 h from time of administration.

A postoperative hand infection may lead to a permanent reduction of hand function, affecting the patient’s quality of life and ability to work [1]. The role of antimicrobial prophylaxis in shorter, clean soft tissue hand surgical procedures is debatable, but it is standard in hand surgeries involving bone, remaining devitalized tissue, and/or implants due to increasing bacterial virulence [2,3]. However, due to the risk of adverse events adequate administration is vital. To assist the immune system in defending the obligate intraoperative wound contamination, prophylactic antimicrobials should attain tissue concentrations higher than the minimum inhibitory concentration (MIC) of likely bacteria from the time of skin incision and at least until wound closure [4-6].

A single preoperative administration of 1,500 mg cefuroxime has shown sufficient sufficient time of the free concentration above the minimal inhibitory concentration (*f*T>MIC) 4 μg/mL for around 5 h for bone, muscle, and subcutaneous tissues of the foot [7]. However, tissue concentrations of cefuroxime have been shown to be heterogeneous across anatomic regions, tissue compartments, and patient groups [7-9]. In hand surgery, the distribution of cefuroxime to relevant target tissues is unknown, as is the optimal time for administration prior to surgery and the potential need for readministration during longer surgeries.

The aim of this randomized controlled trial was to evaluate cefuroxime *f*T>MIC 4 μg/mL in bone (primary effect parameter), the synovial sheath, and subcutaneous tissue of the hand in patients undergoing trapeziectomy following administration of either a single dose of 1,500 mg cefuroxime or 2 repeated doses of 1,500 mg with a 4-h interval. We hypothesized longer *f*T>MIC 4 μg/mL after repeated administration compared with single cefuroxime administration based on an 8-h dosing interval, enabling evaluation of readministration and optimal time for initial administration.

## Methods

### Study design

The study was conducted in the Department of Orthopedic Surgery, Aarhus University Hospital, Denmark. The analyses were performed in the Department of Clinical Biochemistry, Aarhus University Hospital, Denmark. The CONSORT reporting recommendations were followed.

### Participants

16 patients were recruited between August 2019 and May 2021 from the hand outpatient clinic at Aarhus University Hospital by 2 hand surgeons. The inclusion and exclusion criteria are presented in Table 1. During the Covid-19 pandemic, patients were not included and operated on.

**Table 1:** Inclusion and exclusion criteria

**Inclusion criteria**
Scheduled for trapeziectomy at the Department of Orthopedic Surgery, Aarhus University Hospital Age ≥ 18 years Normal blood test (alanin-aminotranferase [ALAT] male 0–70 U/L, female 0–45 U/L, alkaline phosphatase 0–105 U/L, bilirubin 0–25 μmol/L, and creatinine male 60–105 μmol/L, female 45–90 μmol/L For fertile women, use of contraceptive or negative urine human chorionic gonadotropin Written informed consent
**Exclusion criteria**
Diabetes Allergy to beta-lactams (penicillin, cephalosporin, and carbapenem) Previous surgery or fracture in the first metacarpal on the side of surgery Previous vascular surgery on arm (operating side) Antimicrobial treatment with cefuroxime within the 4 days leading up to surgery

### Endpoints

The primary endpoint was cefuroxime *f*T>MIC. Sufficient prophylactic coverage in target tissues was defined as time periods with a concentration above 4 μg/mL. Cefuroxime is a time-dependent antimicrobial, and thus its effect is best evaluated as the time the free drug concentration remains above relevant MIC values. For cefuroxime, the epidemiological cut-off (ECOFF) for the most common etiology of postoperative hand infections, S. aureus, in planktonic form is 4 μg/mL, which represents the MIC cut-off value for susceptible strains [2,10-12]. The secondary endpoints were area under the time-concentration curve (AUC_0–8h_), mean maximal concentration (C_max_), half-life (T_1/2_), and time until C_max_ (T_max_).

### Sample size calculation

In a biopsy study following the administration of 3,000 mg cefuroxime (n = 25), a mean concentration of 49 μg/mL (standard deviation [SD] 22) was found in cancellous bone 45 min after administration [13]. At an equivalent time point following the administration of 1,500 mg (n = 9), the mean cancellous bone concentration based on microdialysates was 18 μg/mL (SD 9) [9]. Using the largest SD (22) across the 2 studies, and a two-sample mean comparison t-test, a significance level of 5%, and a power of 80%, a sample size of 7 patients in each group was needed to demonstrate differences in cancellous bone concentrations between the 2 dosing regimens. To account for the potential risk of technical problems and dropouts, 8 patients were included in each group.

### Description of surgery

Prior to surgery, all patients received a supraclavicular nerve block. Preoperatively, 1,000 mg of cloxacillin (monoclox, Medochemie Ltd, Limassol, Cyprus) was administered with a mean time of 180 min prior to start of microdialysis. An upper arm tourniquet (250 mmHg) was used for all procedures and inflated prior to incision. All surgeries were performed by 2 experienced hand surgeons. They performed the first operation together to attain consensus regarding the technique. ARJ was present at the placement of all catheters to make sure they were placed similarly. Trapeziectomies were performed using a 3 cm dorsolateral incision and the base of the 1st metacarpal was stabilized with a suspensionplasty where a slip of the abductor pollicis longus tendon was looped around the flexor carpi radialis longus tendon and fixed to the base of the first metacarpal with a resorbable suture anchor (MiniLok Quickanchor Plus 2-0, DePuy, Raynham, MA, USA).

### Experiment

**Figure 1:**
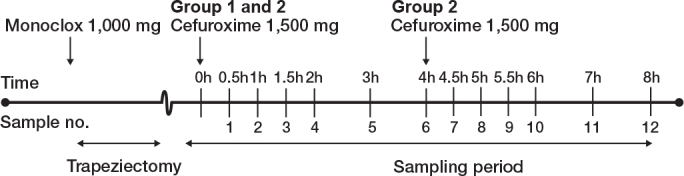
Study time plan. Created with BioRender.com

The experiment was done post-surgery. Administration of cefuroxime marked the start (time zero) of the 8-h sampling period (Figure 1). Prior to administration, each catheter was allowed a minimum of 30 min equilibration. Cefuroxime was administered intravenously over 10 min.

**Figure 2:**
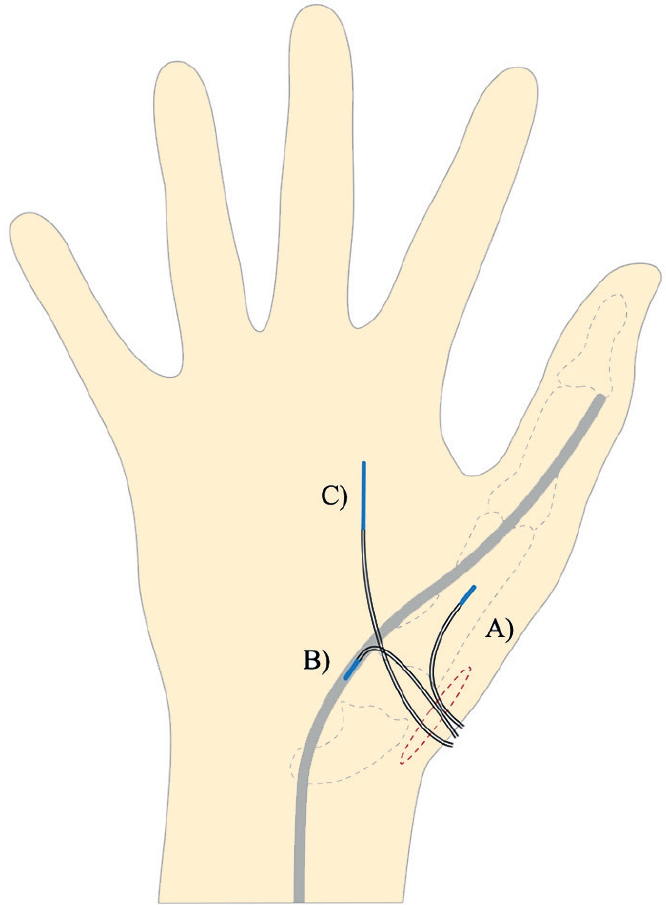
Placement of the microdialysis catheters through the skin incision (red dashed circle). A. 10-mm catheter in the first metacarpal bone. B. 10-mm catheter in the synovial sheath of the extensor pollicis longus tendon. C. 30-mm catheter in the subcutaneous tissue.

When the surgical procedure was performed the catheters were positioned. A drill hole (Ø 2 mm, length 15 mm) was made in the first metacarpal bone using a K-wire and an oscillating technique for placement of a 10 mm microdialysis catheter (M Dialysis, Stockholm, Sweden). Another 10 mm catheter was placed distal to proximal in the synovial sheath of the extensor pollicis longus tendon with an entry point 1 cm dorsal to the skin incision. This was done by making a small incision in the synovial sheath and keeping the entry open by flushing it with saline until placement of the catheter. On the dorsal side of the hand from the skin incision and in the distal direction, a 30-mm catheter was placed in the subcutaneous tissue using an introducer (Figure 2). The surgical wound was closed using Vicryl 3-0 in the capsule and a Nylon 6-0 running suture in the skin. The microdialysis catheters were fixed to the skin with a transparent film bandage (Kliniderm, Medeco BV, Oud-Beijerland, the Netherlands), and a soft dressing was applied. Finally, the location of the metacarpal catheter in the bone was confirmed by perioperative fluoroscopy.

### Randomization

Postoperatively, each patient was randomized to either Group 1 (1 x 1,500 mg cefuroxime, n = 8) or Group 2 (2 x 1,500 mg cefuroxime, n = 8). Group allocation was performed by opening a randomly drawn opaque envelope from a larger non-translucent envelope containing 16 envelopes that were set up prior to the initiation of the study.

### Microdialysis

Microdialysis is a diffusion-based sampling method allowing for continuous sampling of an unbound drug from various target tissues simultaneously [14,15]. In this study, individual catheter calibration was performed with the internal calibrator, meropenem (concentration in perfusion fluid; 7 μg/mL).

The microdialysis equipment was obtained from M Dialysis AB (Stockholm, Sweden). The CMA 107 perfusion pumps were set at a flowrate of 2 μL/min, and type 63 catheters (10 and 30 mm) were used.

### Sampling

A total of 12 dialysates and 12 venous samples were collected over the 8-h observation period. Dialysates were collected from each of the 3 microdialysis catheters at 30, 60, 90, 120, 180, 240, 270, 300, 330, 360, 420, and 480 min. Venous samples were collected at the midpoint of each sampling interval from a cubital vein catheter in the non-operated arm.

### Handling of samples

Immediately after collection, venous samples were stored at 3–5°C for a maximum of 8 h before being centrifuged for 10 min at 3,000 g at 5°C. Plasma was stored at –80°C until analysis. All dialysates were stored on dry ice for a maximum of 8 h before being transferred to a –80°C freezer until further analysis.

### Ultra-high-performance liquid chromatography analysis

The concentrations of cefuroxime and meropenem in plasma and dialysates were quantified using a validated ultra-high-performance liquid chromatography assay [16]. The inter-run imprecisions (percent coefficients of variation [% CV]) were 4.3% (4.7%) at 2.5 μg/mL and 1.6% (6.2%) at 38 μg/mL for the quantification of cefuroxime and 3.0% at 2.0 μg/mL for the quantification of meropenem. The accuracy of the assay was evaluated by repeated measurements of 5 different cefuroxime formulations and was found to be between –3.3% and 5.8%. The lower limits of quantification were 0.006 μg/mL (cefuroxime) and 0.5 μg/mL (meropenem) [17].

### Pharmacokinetic analysis and statistics

All dialysate concentrations were ascribed to the midpoint of the sampling interval. For each patient and each compartment, the primary endpoint T>MIC 4 μg/mL was estimated by linear interpolation in Microsoft Excel (version 16, Microsoft Corp, Redmond, WA, USA). The time until attainment of a mean concentration of 4 μg/mL and the time until a decrease below 4 μg/mL were calculated. For Group 2 (2 x 1,500 mg), only the first time until a decrease below a mean concentration of 4 μg/mL was noted. In Stata (version 16.0, StataCorp LLC, College Station, TX, USA) the secondary endpoints were calculated for each patient and each compartment: AUC, C_max_, T_1/2_, and T_max_. AUC was determined by the linear up–log down trapezoidal method. For Group 1 (1 x 1,500 mg), C_max_ and T_1/2_ were calculated for the entire sampling period (0–8 h), while for Group 2 (2 x 1,500 mg) they were calculated only for the first sampling interval (0–4 h). T_1/2_ was calculated using the formula T_1/2_ = ln(2)/ λ_eq_, where λ_eq_ is the terminal-elimination-rate constant estimated by linear regression of the log concentration time. T_max_ was calculated as the time until C_max_. A mixed model for repeated measurements with compartments as fixed effect and patient identification as random effect was applied. With an F-test, overall comparisons between the compartments were done, while a t-test was applied for pairwise comparison. The model assumptions were tested by visual diagnosis of residuals, fitted values, and estimates of random effects. Means were given with either SD or 95% confidence interval (CI).

### Ethics, registration, data sharing plan, funding, and disclosures

The study was approved by the Danish Medicines Agency (EudraCT number 2019-001134-33), the Central Denmark Region Committees on Health Research Ethics (Registration number 1-10-72-55-19), and the Danish Data Protection Agency (Registration number 1-16-02-104-19). The study was conducted in accordance with the declaration of Helsinki and the ICH harmonized tripartite guidelines for good clinical practice. Mandatory monitoring was performed by the Good Clinical Practice Unit at Aalborg and Aarhus University Hospitals. Anonymized data of this study may be available from the corresponding author upon reasonable request. This work was supported by grants from the Merchant L.F. Foghts Foundation. The funding source did not play a role in the study or publication. On behalf of all authors, the corresponding author states that there is no conflict of interest. Complete disclosure of interest forms according to ICMJE are available on the article page, doi: 10.2340/17453674.2024.41343

## Results

**Figure 3:**
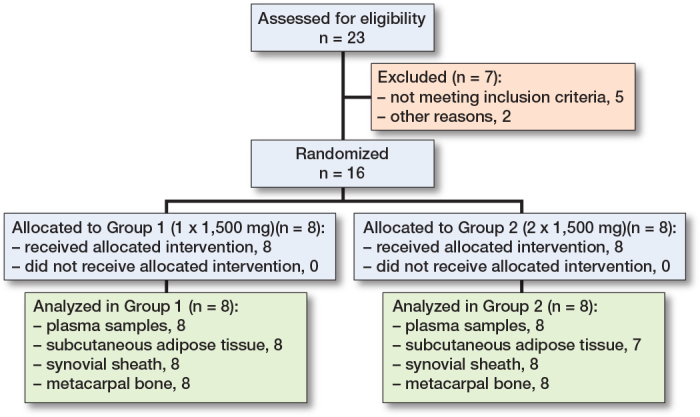
Patient flowchart.

A total of 23 patients were assessed for eligibility and 7 pateints were excluded, giving a total of 16 patients (Figure 3 and Table 2). In both groups there was a preponderance of female participants. All 16 included patients completed the study without any adverse events such as reaction to medication and/or complication with microdialysis catheter removal. One subcutaneous tissue catheter in Group 2 malfunctioned due to membrane damage. The mean relative recovery rates (SD) were 25% (7) for the metacarpal bone, 31% (6) for the synovial sheath, and 44% (9) for the subcutaneous tissue.

**Table 2:** Patient demographics. Values are mean (SD) unless otherwise specified

Variable	Group 1	Group 2
Number of patients, n	8	8
Sex ratio (female/male), n	6/2	7/1
Operated hand ratio (left/right), n	4/4	3/5
Age (years)	66 (9)	63 (8)
Weight (kg)	70 (11)	81 (21)
Height (cm)	168 (10)	168 (6)
Plasma creatinine (μmol/L)	69 (17)	63 (7)
Plasma ALAT (μmol/L)	22 (10)	24 (10)
Plasma bilirubin (μmol/L)	8 (3)	11 (3)
Plasma alkaline phosphatase (U/L)	77 (18)	77 (20)
Operation time (including catheter placement and bandaging) ^**a**^	76 (17)	85 (19)
Tourniquet application duration ^**a**^	73 (12)	82 (18)
Time to cefuroxime administration after tourniquet release ^**a**^	48 (14)	61 (24)

a in minutes

### Prophylactic antimicrobial coverage time

**Figure 4:**
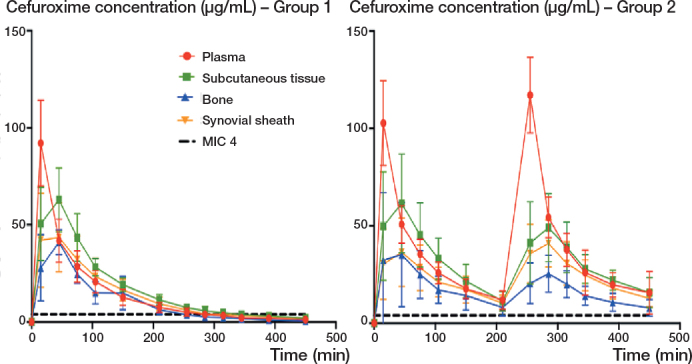
Mean concentration-time profiles for Group 1 (1 × 1,500 mg) and Group 2 (2 × 1,500 mg) for plasma, subcutaneous tissue, bone, and synovial sheath. Bars represent CI. MIC: minimal inhibitory concentration.

The *f*T>MIC of 4 μg/mL was found to be significantly longer in the metacarpal bone in Group 2 compared with Group 1, with a mean difference of 199 min (CI 158–239). Overall, the mean (%) cefuroxime *f*T>MIC 4 μg/mL in all tissue compartments was longer in Group 2 (range 96–100%) than in Group 1 (range 52–75%) for the 8-h sampling period (Figure 4 and Table 3).

**Table 3:** *f*T>MIC 4 μg/mL for plasma, subcutaneous adipose tissue, synovial sheath, and bone presented as mean minutes [CI], and (%) of sampling time for Group 1 (1 x 1,500 mg) and Group 2 (2 x 1,500 mg)

Factor	n	Group 1 mean [CI] (%)	n	Group 2 mean [CI] (%)	Differences mean [CI]
Plasma	8	280 [251–308] (62)	8	449 [421–478] (100)	170 [129–210]
Subcutaneous adipose tissue	8	336 [308–365] (75)	7	445 [414–475] (99)	108 [66–150]
Synovial sheath	8	315 [287–344] (70)	8	446 [418–475] (99)	131 [90–171]
Bone	8	235 [206–263) (52)	8	433 [405–462] (96)	199 [158–239]

### Time until concentration above and below sufficient prophylactic antimicrobial coverage

A mean cefuroxime concentration of 4 μg/mL was reached for all compartments for both groups within a mean time of 6 min (range 0–27 min) (Table 4). In Group 1 (1 x 1,500 mg), the cefuroxime concentration in all compartments decreased below a value of 4 μg/mL after a mean time of 5 h 38 min (Table 4). The shortest time until *f*T<MIC 4 μg/mL was seen in bone in Group 1, with a mean time of 3 h 59 min (range 3 h 2 min to 4 h 43 min) (Table 3). In Group 2 (2 x 1,500 mg), only 4 patients, and only in the bone compartment, experienced a drop below a concentration of 4 μg/mL, resulting in a mean time with a concentration above 4 μg/mL of 6 h 50 min (range 3 h 25 min to 7 h 30 min). For the remaining tissue compartments in Group 2, the mean concentration remained above 4 μg/mL for the entire observation period (Table 4).

**Table 4:** Mean time in minutes (range) until the cefuroxime concentration exceeded 4 μg/mL and mean time in minutes (range) for a decrease below a concentration of 4 μg/mL for Group 1 (1 x 1,500 mg) and Group 2 (2 x 1,500 mg)

Factor	Group 1		Group 2		Differences mean [CI]
	n	mean (range)	n	mean (range)	
Mean time until mean concentration > 4 μg/mL					
Plasma	8	1 (0–1)	8	1 (1–1)	–0.1 [–4 to 4]
Subcutaneous adipose tissue	8	2 (1–3)	7	5 (1–27)	4 [–1 to 48]
Synovial sheath	8	2 (1–5)	8	4 (1–11)	2 [–3 to 46]
Bone	8	4 (1–12)	8	6 (1–19)	2 [–2 to 47]
Mean time until mean concentration < 4 μg/mL					
Plasma	8	279 (176–368)	8	450 (450–450)	171 [121 to 220]
Subcutaneous adipose tissue	8	338 (244–406)	7	450 (450–450)	112 [61 to 164]
Synovial sheath	8	318 (226–434)	8	450 (450–450)	132 [83 to 182]
Bone	8	239 (187–284)	8	410 (205–450)	171 [121 to 221]

### Pharmacokinetic parameters

Values for AUC, C_max_, T_max_, and T_1/2_ are presented in Table 5. With the exception of bone with a mean difference of 2.9 min x 10^3^ μg/mL (CI –0.5 to 6.4), there was a significant longer AUC in Group 2 compared with Group 1.

**Table 5:** Pharmacokinetic parameters for plasma, subcutaneous adipose tissue, synovial sheath, and bone for Group 1 (1 x 1,500 mg) and Group 2 (2 x 1,500 mg)

Pharmacokinetic parameters	n	Group 1	n	Group 2	Difference [CI]
AUC, mean min x 10^3^ μg/mL [CI]					
Plasma	8	6.7 [4.2–9.1]	8	16.5 [14.1–19.0]	9.8 [6.4 to 13.3]
Subcutaneous t.	8	8.0 [5.5–10.4]	7	14.3 [11.7–16.8]	6.3 [2.7 to 9.8]
Synovial sheath	8	6.3 [3.8–8.7]	8	10.6 [8.2–13.1]	4.4 [0.9 to 7.8]
Bone	8	4.8 [2.3–7.2]	8	7.7 [5.3–10.2]	2.9 [–0.5 to 6.4]
C_max_, mean μg/mL [CI] ^**a**^					
Plasma	8	92 [74–111]	8	94 [75–112]	2 [–24 to 28]
Subcutaneous t.	8	64 [45–82]	7	65 [46–85]	1 [–26 to 28]
Synovial sheath	8	46 [28–65]	8	37 [18–55]	–10 [–36 to 16]
Bone	8	44 [25–62]	8	40 [22–59]	–3 [–29 to 23]
T_max_, mean min (range)					
Plasma	8	15 (0–30)	8	23 (7–38)	8 [–14 to 29]
Subcutaneous t.	8	38 (22–53)	7	45 (29–61)	7 [–15 to 29]
Synovial sheath	8	38 (22–53)	8	60 (45–75)	23 [1 to 44]
Bone	8	41 (26–57)	8	62 (47–77)	21 [–1 to 42]
T_1/2_, mean min [CI] ^**a**^					
Plasma	8	57 [46–68]	7	61 [50–73]	4 [–12 to 20]
Subcutaneous t.	8	86 [75–97]	6	82 [70–95]	–4 [–20 to 13]
Synovial sheath	8	93 [82–104]	8	99 [86–112]	6 [–11 to 22]
Bone	8	77 [66–88]	4	76 [61–92]	–1 [–20 to 18]

a For Group 2, C_max_ and T_1/2_ are calculated based on only the first dosing interval.

AUC: area under the concentration-time curve, Subcutaneous t. = Subcutaneous adipose tissue, C_max_: peak drug concentration, T_max_: time until C_max_, T_1/2_: half-life.

## Discussion

We aimed to evaluate cefuroxime *f*T>MIC 4 μg/mL in bone, the synovial sheath, and subcutaneous tissue of the hand in patients undergoing trapeziectomy following administration of either a single dose of 1,500 mg cefuroxime or 2 repeated doses of 1,500 mg with a 4-h interval. The study showed that, in hand surgery, a single administration of 1,500 mg of cefuroxime can be expected to provide sufficient coverage in all the investigated tissues for a mean duration of 3 h 59 min after administration. A 2nd dose after 4 h prolongs *f*T>MIC (4 μg/mL) to 6 h 50 min. For longer hand surgeries involving bone, cefuroxime should be readministered after 3 h 59 min for sufficient coverage. Sufficient mean target tissue concentrations were achieved swiftly (mean 6 min), indicating that preoperative cefuroxime administration close to the surgical incision site is safe. A recent clinical perioperative cefuroxime microdialysis study investigating bone and subcutaneous tissue cefuroxime (1,500 mg) concentrations in patients undergoing foot surgery found similar penetration, with sufficient coverage within 23 min [7]. However, to ensure sufficient penetration in all compartments and individuals, it may be reasonable to apply the lowest value in the range rather than the mean value, suggesting that administration should be performed 27 min prior to incision.

### Pharmacokinetic target

We investigated which dosing regimen resulted in the longest *f*T>MIC. However, the needed *f*T>MIC, especially in prophylactic settings, can be debated as the effective *f*T>MIC is often evaluated for infections [18]. A short *f*T>MIC might be sufficient to assist the immune system in preventing surgical site infections in hand surgery (prophylaxis), but this necessitates a different study design with a much larger study population and follow-up for infection. Additionally, the target value in the present study was set at 4 μg/mL (ECOFF). Many S. aureus strains are susceptible to lower MIC, resulting in longer *f*T>MIC if lower MIC values are evaluated [10]. Yet, for perioperative prophylaxis encompassing the variety of most patients, it may be reasonable to aim for ECOFF to protect against the majority of susceptible bacteria.

### Choice of administration type and dose

A higher risk of postoperative hand infection has been found for surgeries lasting longer than 2 h [2,19]. This may be reasoned by a compromised microenvironment caused by the prolonged surgery impairing the effect of the immune system. In these cases, adequate antimicrobial prophylaxis may be particularly important. In the present study, the mean surgery time was 1 h 20 min, and thus a single bolus of 1,500 mg cefuroxime would theoretically suffice. An alternative to bolus administration is continuous infusion, which can ensure sufficient coverage throughout long-lasting surgeries without the need for readministration. However, it is crucial to ensure an initial bolus administration that will attain a sufficient concentration, which can then be maintained by a continuous infusion. In a study on total knee replacement patients, continuous infusion was found to be superior compared with a standard bolus in terms of tissue *f*T>MIC, even after administration of equal doses [9]. The application of higher cefuroxime doses has also been considered to improve *f*T>MIC. However, a previous porcine study evaluating concentrations in plasma, subcutaneous tissue, knee joint, cortical and cancellous bone showed only minimal benefit in terms of *f*T>MIC 4 μg/mL for a single intravenous administration of 3,000 mg cefuroxime compared with 1,500 mg [17]. Additionally, dosage adjusted to patient weight should also be considered [20].

### Synovial sheath

This is the first study to sample cefuroxime concentrations in the synovial sheath using microdialysis. The application of microdialysis in the synovial sheath is simple and minimally invasive, which may motivate future pharmacokinetic synovial sheath evaluations of different antimicrobial drugs and administration forms. Pyogenic tenosynovitis is a common acute condition threatening hand function, but evidence regarding optimal antimicrobial regimens is still lacking [21]. In the present study, sampling was done in non-infected tissues, showing the penetration of systemic antimicrobials in the tendon sheath. However, antimicrobial penetration to infected tissues is lower than in healthy tissues, and thus future studies investigating target concentrations during pyogenic synovitis are needed [22,23].

### Limitations

The main limitation of the present study was that cefuroxime was administered postoperatively and after tourniquet release. Therefore, the observed pharmacokinetic profile of cefuroxime may differ from the true perioperative setting. However, in the foot, only a minor negative perioperative effect and a positive postoperative effect of the tourniquet have been found on local tissue cefuroxime concentrations [7]. The use of tourniquet on an upper extremity is not expected to lead to different results. The patient demographics were representative of most patients with degenerative joint conditions seen in a hand clinic. The sample size was small, but due to dense microdialysis sampling it was sufficient to describe group differences.

### Conclusion

The *f*T>MIC proved to be significantly longer in the metacarpal bone in Group 2 compared with Group 1, with a mean difference of 199 min. This tendency was also found in the remaining compartments. A single administration of 1,500 mg of cefuroxime provided antimicrobial tissue coverage in subcutaneous tissue, the synovial sheath, and bone tissue of the hand for a minimum mean time of 3 h 59 min, which also indicates the time when readministration is required in longer surgeries. Sufficient concentrations were attained within a mean of 6 min. However, to achieve sufficient concentrations in all individuals, the administration of cefuroxime should be performed a minimum of 27 min prior to incision. 

## Notes

ARJ, PH, MB, NPJ, and MS initiated and designed the study. ARJ, CHA, and MS conducted the surgery and placed all the catheters. ARJ and PH collected the data. Statistical analysis and interpretation of data were performed by ARJ, PH, MB, and MS. All authors drafted and revised the manuscript.

The authors would like to thank the Department of Orthopaedic Surgery, Aarhus University Hospital, and the Orthopaedic Research Unit, Aarhus University Hospital, for supporting this study. They also thank biomedical laboratory scientist Anette Baatrup for her help with the analyses. Most importantly, they express their deep gratitude to the patients who agreed to take part in the study.

Handling co-editors: Bart A. Swierstra and Philippe Wagner

Acta thanks Peter D Croughs and Gerald A Kraan for help with peer review of this manuscript.
